# Use of Preabortion Ultrasonography Among Telehealth Medication Abortion Patients

**DOI:** 10.1016/j.whi.2025.06.002

**Published:** 2025-07-23

**Authors:** Sylvie T. Wilson, Lisa Peters, Leah R. Koenig, Suzanne O. Bell, Ushma D. Upadhyay

**Affiliations:** a Department of Population, Family, and Reproductive Health, Johns Hopkins Bloomberg School of Public Health, Baltimore, Maryland; b Department of Obstetrics, Gynecology, and Reproductive Sciences, University of California, San Francisco, Oakland, California; c Department of Epidemiology and Biostatistics, University of California, San Francisco, San Francisco, California; d Center for Gender and Health Justice, University of California Global Health Institute, Oakland, California

## Abstract

**Background:**

Since 2020, some clinicians have offered telehealth medication abortion care that does not require ultrasonographic screening for eligible patients. However, some telehealth medication abortion patients nonetheless obtain ultrasonography.

**Objective:**

This study aims to understand which patients obtain ultrasonography before telehealth medication abortion, their reasons for ultrasonography, and where they obtain it.

**Study Design:**

Using data from the California Home Abortion by Telehealth (CHAT) Study, a cohort study of patients using telehealth abortion services in 2021 and 2022, participants were asked whether they had pre-abortion ultrasonographic imaging, reasons for the ultrasonography, and where they obtained it. We analyzed these responses using chi-squared tests, Fisher’s exact tests, and multivariable logistic regression.

**Results:**

Among 1,775 participants, 11% (*n* = 194) obtained ultrasonography before their abortion. In the multivariable model, participants who were younger, were food insecure, had a pregnancy duration of 35 days (5 weeks) or more, or were unsure of their pregnancy duration were significantly more likely to obtain pre-abortion ultrasonography. The most common reason for ultrasonography was to assess pregnancy duration (69%). Most participants obtained imaging at a clinic (41%) or at an emergency room or hospital (27%), although some obtained imaging at crisis pregnancy centers (14%). Of those who obtained ultrasonography, 8% were referred by their telehealth provider; the remainder sought ultrasonography on their own initiative.

**Conclusion:**

Hospitals, clinics, and imaging facilities can create policies to increase the accessibility of ultrasonography for those who desire or require pre-abortion ultrasonography.

Medication abortion most commonly involves the use of mifepristone and misoprostol or misoprostol alone to end a pregnancy and is typically prescribed for people with pregnancies up to 77 days gestation. Clinicians have historically used ultrasonographic imaging to confirm the pregnancy is in the uterus, rule out ectopic pregnancy, and determine the duration of a pregnancy before providing patients with abortion medications (Raymond et al., 2018). However, the COVID-19 pandemic led some clinicians to forgo routine ultrasonography before providing abortion medications to minimize physical contact (Chong et al., 2021; Upadhyay et al., 2020). Instead, many clinicians have been screening patients for risk of ectopic pregnancy and pregnancy duration by medical history alone (Upadhyay, Raymond, et al., 2022). This strategy allows eligible patients to access medication abortion pills via telehealth and mail without any in-person screening tests (Raymond et al., 2020). Evidence indicates this approach is safe and acceptable and very rarely (0.5%) results in serious adverse events (Chong et al., 2021; Kerestes et al., 2021; Upadhyay, Raymond, et al., 2022; Upadhyay, Koenig, et al., 2024). The Society of Family Planning, the American College of Obstetricians and Gynecologists, and the National Abortion Federation endorsed this change in clinical practice as of October 2020 (Raymond et al., 2020; Committee on Practice Bulletins—Gynecology Society of Family Planning, 2020; National Abortion Federation, 2024). As a result, patients with no risk factors for ectopic pregnancy who are relatively certain of the date of their last menstrual period or otherwise know their pregnancy duration is less than 11 or 12 weeks may be prescribed medication abortion pills without ultrasonographic imaging.

The updated guidelines allow many patients to receive a telehealth medication abortion without ultrasonography or other in-clinic tests based on their medical history and response to screening questions (Raymond et al., 2018; Committee on Practice Bulletins—Gynecology Society of Family Planning, 2020). Patients who are not certain of the date of their last period and those who are at risk for ectopic pregnancy are generally not eligible for no-test telehealth abortion care and may be referred to obtain ultrasonographic imaging before obtaining a medication abortion. Among those who are eligible for no-test telehealth abortion, little is known regarding who obtains pre-abortion ultrasonographic imaging in the United States and why. This information is salient because obtaining pre-abortion ultrasonographic imaging that is not medically indicated may increase travel time, increase the cost of overall abortion care, decrease convenience for patients, and ultimately delay a patient’s receipt of abortion (Koenig et al., 2023).

This paper examines the factors associated with obtaining pre-abortion ultrasonography, reasons for obtaining it, and the locations from which they were obtained in a population of patients seeking telehealth medication abortion in the United States. Increasing our understanding of patient perspectives around ultrasonography can help to shape abortion services, guide institutional policy, and shape clinical practice guidelines.

## Materials and Methods

We conducted a secondary data analysis of data from the California Home Abortion by Telehealth (CHAT) Study to understand associations between sociodemographic characteristics, reproductive health history, and experience of pre-abortion ultrasonography. The CHAT Study aimed to understand the safety, effectiveness, and acceptability of telehealth abortion care provided by three virtual clinics in the United States operating across 20 states and the District of Columbia (Abortion on Demand, Hey Jane, and Choix) (Koenig et al., 2023; 2024; Upadhyay et al., 2024). Data for the study included deidentified electronic medical record data for all abortions performed at the participating virtual clinics between April 2021 and January 2022 and data from three surveys administered online. Patients provided informed consent for the virtual clinics to share their electronic medical record data with the CHAT study team before starting each survey. The University of California, San Francisco Institutional Review Board provided ethical approval (#20-32951).

The three surveys included a baseline survey at abortion intake, a survey 1 week after intake, and a final survey conducted 4 weeks after intake. The surveys were shared with participants until approximately 400 participants from each clinic completed all three surveys. Participants were compensated with $50 after they completed all three surveys. Most questions used in this study were from the baseline survey. We describe the methodology of the CHAT Study in greater detail elsewhere (Koenig et al., 2024). Not all participants who shared electronic medical record data took part in the baseline survey. Conversely, some patients who took part in the baseline survey did not have electronic medical record data available. Participants were included in our analysis if they consented to share electronic medical data, completed the baseline survey, had complete ultrasonography data, and fully completed the questions in the baseline survey regarding if and where they obtained ultrasonographic imaging.

The baseline survey collected participant sociodemographic characteristics and pregnancy history, whether the person obtained ultrasonography, and, if so, the reason for obtaining it. The survey provided a list of reasons for obtaining ultrasonographic imaging, including “not sure how many weeks pregnant I was,” “I wanted to confirm how many weeks pregnant I am,” “I went to a clinic or hospital for other reasons and they did an ultrasound,” “I wanted to receive care in a clinic,” “I was worried about ectopic pregnancy,” “I wanted to see the ultrasound picture,” “I was considering keeping the pregnancy,” or “other,” with the opportunity to define a reason not listed. Participants who selected “not sure how many weeks pregnant” and “wanted to confirm weeks pregnant” we grouped into a new category: “to assess pregnancy duration.” Responses of those who selected “other” were recategorized into one of the existing categories or a new category was developed. New categories included “wanted to confirm pregnancy after a positive at home pregnancy test” and “experienced pregnancy symptoms.”

The closed-ended list of locations included clinic (abortion, family planning, primary care), obstetrician or gynecologist or primary care provider, imaging facility, emergency department or hospital, radiology department, crisis pregnancy center, another location (other), or unknown. We recategorized other responses into one of the existing categories or unspecified clinic. If the location of the ultrasonography was missing in the survey data, we used the ultrasonography location documented in the electronic medical record data if available. Participants were able to select multiple reasons for obtaining ultrasonographic imaging, or multiple locations they visited for ultrasonography.

The baseline survey also asked participants about their sociodemographic characteristics including age (birth date, age, neither) and race/ethnicity (American Indian or Alaska Native, Asian, Black or African American, Hispanic or Latinx/a/o, Middle Eastern or North African, Native Hawaiian or other Pacific Islander, white, other, prefer not to answer, and do not know), student status (in school full time, in school part time, not in school), education (eighth grade or less, some high school, high school diploma or GED, some college or technical school but no degree, associate’s degree, bachelor’s degree, graduate and professional degree), employment status (employed full time [32 or more hours a week], employed part time [less than 32 hours a week], self-employed, unemployed and looking for work, not working and not looking for employment, homemaker, military, unable to work, and other), and food insecurity (never, sometimes, or often). We included these characteristics as covariates based on their demonstrated relationship with health care–seeking behavior, abortion access, or both (Blakeney et al., 2019; Drewnowsi, 2022; Green, 2018; Hoffman et al., 2008; Sutton et al., 2021). We operationalized these characteristics as categorical variables. Food insecurity was assessed with the following survey items: “in the last month, we worried whether our food would run out before we got money to buy more,” or “in the last month, the food that we bought just didn’t last, and we didn’t have enough money to get any more.” In addition to items from the survey, we analyzed participants’ linked electronic medical record data to identify whether the ultrasonography was due to clinician referral.

We first described sociodemographic characteristics and ultrasonographic imaging care-seeking variables using frequencies and percentages. We then used *χ*^2^ tests and Fisher’s exact tests to understand the association between sociodemographic characteristics and prior reproductive health experiences and their associations with the use of ultrasonography. Missingness indicators were used when participants did not provide responses to survey questions, which allowed us to include data from participants who skipped survey questions and had incomplete data. Next, we conducted a multivariable logistic regression to assess the associations between sociodemographic characteristics and reproductive health experiences with the use of ultrasonographic imaging. Some variables were grouped when conducting the multivariable logistic regression; the Asian, unknown, and other responses in the race category were combined, as were none and unknown responses in the insurance category and none and unknown responses in the prior pregnancies category, all owing to small numbers of individuals obtaining ultrasonographic imagining in these groups. Age groups were combined into younger than 25, 25–34, and 35 years or older. Participants younger than 18 years and between the ages of 18 and 24 were grouped into the younger than 25 group owing to small sample sizes. We also described facility types where participants reported obtaining ultrasonographic imaging and the reasons for obtaining them. Finally, for the subset of survey participants with linked electronic medical records, we examined the proportion of participants who were referred for ultrasonographic imaging by their telehealth clinician.

All analyses were conducted using Stata version 15.1 (College Station, TX). We considered p values of less than .05 to be statistically significant. We used missingness indicators throughout this analysis to address missing data owing to nonresponse to survey items.

## Results

A total of 6,974 patients shared electronic medical record data. Overall, 1,795 participants enrolled in the survey component of the CHAT Study; of these, 1,775 completed the baseline survey, provided responses to the baseline survey questions pertaining to ultrasonographic imaging, and were included in the analytic sample. Within the analytic sample of 1,775 survey participants, 141 did not have linked electronic medical record data.

Approximately 1 in 10 survey participants (*n* = 194 [11%]) obtained ultrasonography before obtaining medication abortion (Table 1). Bivariate associations revealed several significant associations between sociodemographic characteristics and the obtention of ultrasound, including younger age (age < 25 years), fewer years of completed education (high school or less, or some college, technical school, or associate’s degree), being unemployed, experiencing food insecurity, and having a pregnancy duration of 50 days (7 weeks) or more.

The multivariable logistic regression model revealed younger age, employment, food insecurity, and greater pregnancy duration were associated with ultrasonography (Table 2). Individuals aged 25–34 years (adjusted odds ratio [aOR], 0.65; 95% confidence interval [CI], 0.044–0.96) and those 35 years or older (aOR, 0.42; 95% CI, 0.25–0.72) had a significantly lower odds of having ultrasonographic imaging than those younger than age 25. Food insecurity was also a significant predictor of ultrasonography; those who experienced food insecurity sometimes or often were significantly more likely to obtain imaging (aOR, 1.51; 95% CI, 1.08–2.10). Greater pregnancy duration was strongly associated with ultrasonographic screening. Individuals with pregnancy durations of 35–49 days (aOR, 2.98; 95% CI, 1.72–5.17), 50 or more days (aOR, 9.48; 95% CI, 5.32–16.87), or who had an unknown duration (aOR, 4.42; 95% CI, 2.03–9.60) had significantly higher odds of obtaining ultrasonography than those with pregnancy durations of less than 35 days. Participants who answered that their employment status was unknown were also significantly more likely to obtain ultrasonography than those who answered employed or unemployed (aOR, 2.87; 95% CI,1.01–8.15). Race or ethnicity, prior pregnancies, and health insurance status were not significant predictors.

**Figure 1.**
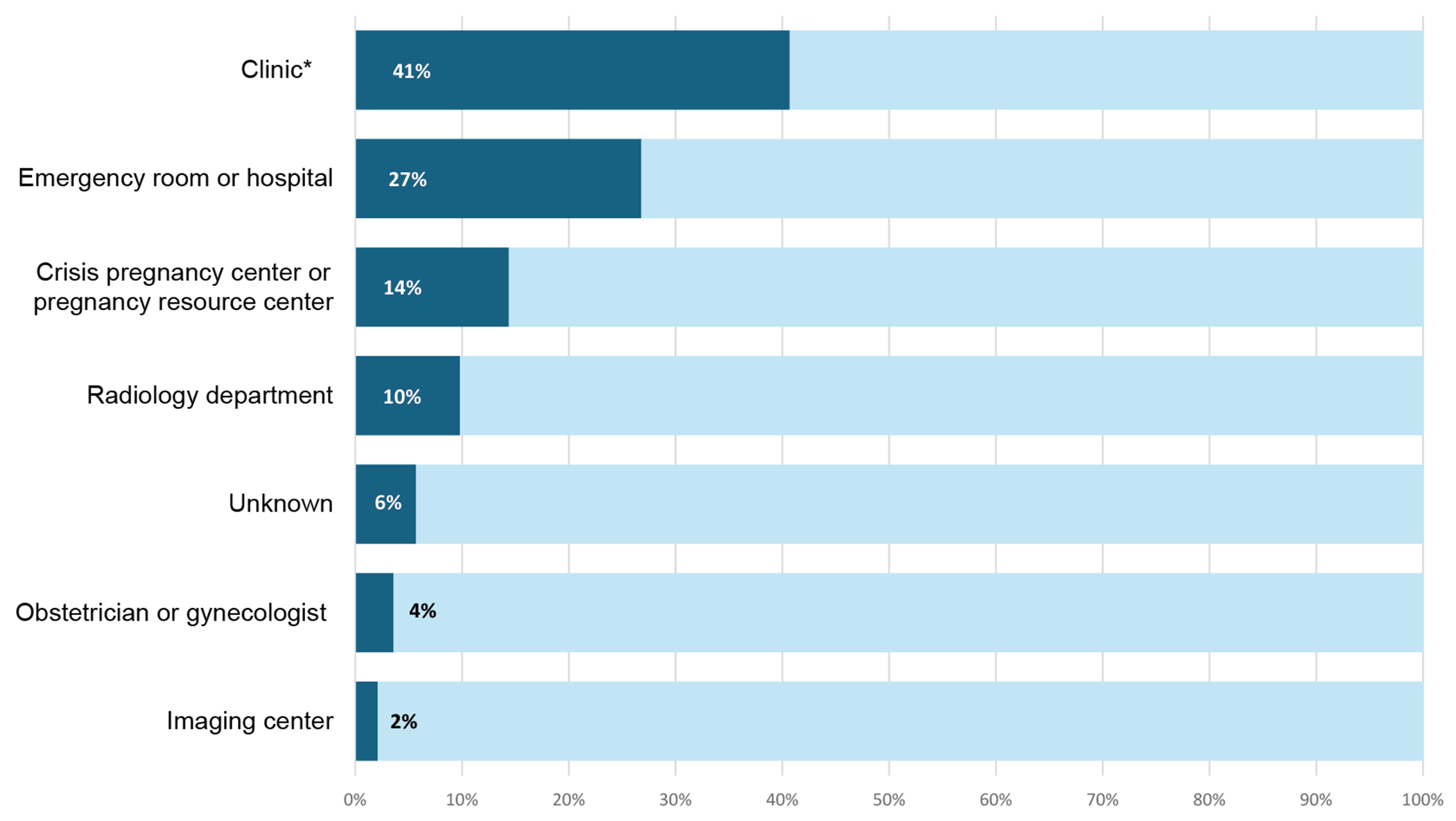
Locations where telehealth abortion patients obtained ultrasonographic imaging in the California Home Abortion by Telehealth Study from 2021 to 2022. Participants could select more than one response (*n* = 194 for each location). *Clinic includes abortion clinic, family planning clinic, primary care clinic and unspecified clinic

Among the 194 participants who reported having ultrasonography, most obtained it at a clinic (41%) (Figure 1). More than one-fourth (27%) obtained ultrasonographic imaging at an emergency room or hospital, 14% at a crisis pregnancy center, 10% at a radiology department, 4% at their obstetrician or gynecologist’s office, and 2% at an imaging center.

**Figure 2.**
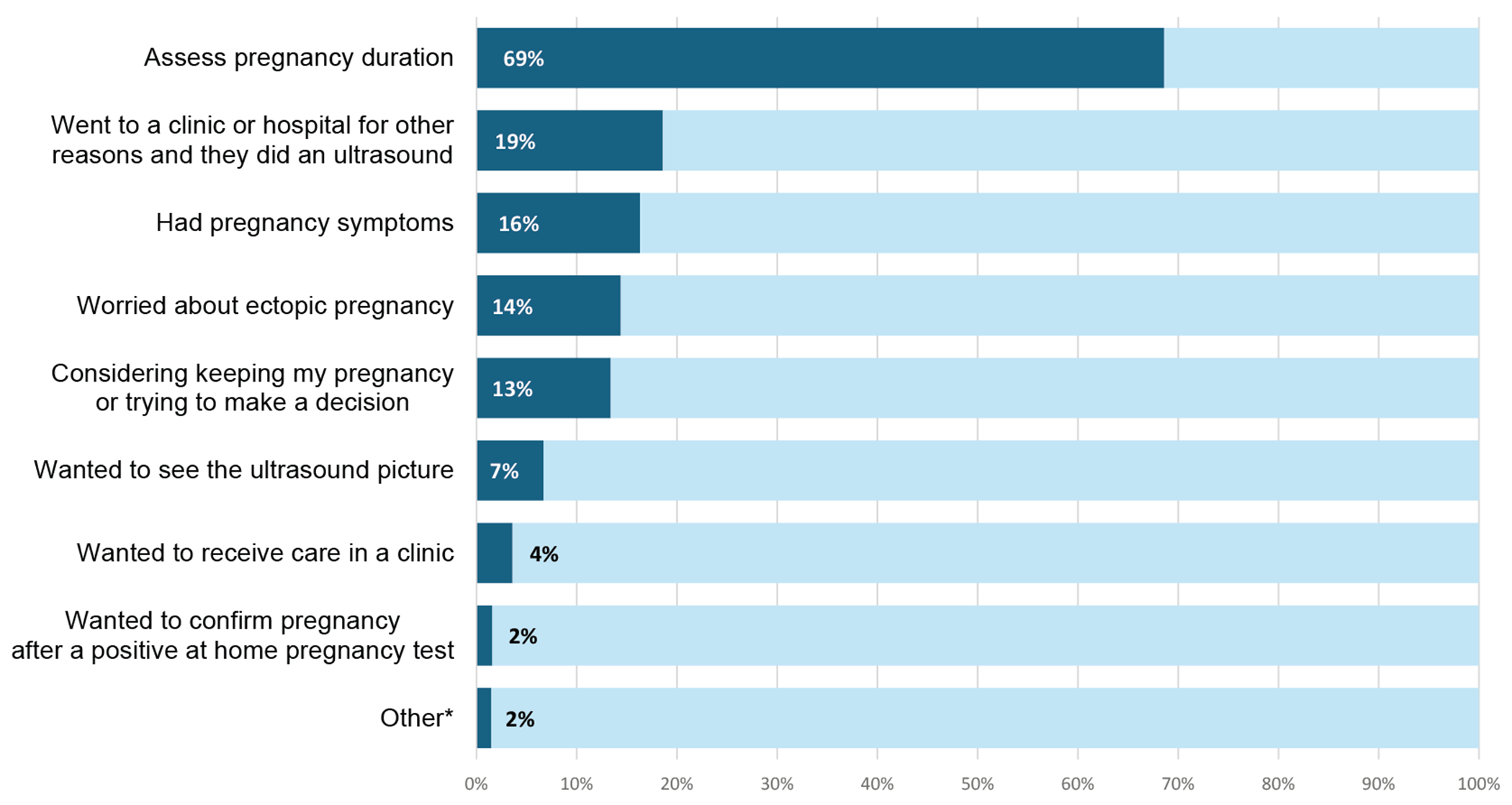
Reasons telehealth abortion patients cited for obtaining an ultrasonographic imaging in the California Home Abortion by Telehealth Study from 2021 to 2022. Participants could select more than one response (*n* = 194 for each reason). *Other included “scared,” “my boyfriend wanted me to keep the child, but I didn’t so wanted to make it look like a miscarriage,” and “no symptoms, I just knew I was pregnant.”

Most participants (69%) who obtained ultrasonography reported that they did so to assess pregnancy duration (Figure 2). Nearly one-fifth (19%) of those who obtained ultrasonographic imaging went to a health care provider for other reasons and were given ultrasonography during their visit. A similar number of participants reported obtaining ultrasonography because they experienced physical symptoms (e.g., cramping, breast tenderness) and wanted to confirm pregnancy (16%), because they had concerns about ectopic pregnancy (14%), or because they were considering continuing their pregnancy (13%).

We analyzed survey participants’ electronic medical record data to understand whether the use of ultrasonographic imaging was primarily driven by clinician recommendation or patient preference. Overall, of the 194 participants who selfreported that they obtained ultrasonography in the baseline survey, 70 did not have linked electronic medical record data. Among the remaining 124 participants, only 10 (8%) were referred for screening ultrasonography by the virtual clinic’s clinician. The remainder sought ultrasonography of their own volition.

## Discussion

Approximately 1 in 10 telehealth abortion patients had ultrasonography, and most of them sought it based of their own volition as opposed to clinician referral. These data suggest that most telehealth medication abortion patients do not obtain ultrasonography. This finding is similar to those of previous studies of pregnant people using online telehealth abortion and is in line with expectations, as ultrasonography is no longer required for most patients using telehealth medication abortion (Anger et al., 2021; Upadhyay, Raymond, et al., 2022; Upadhyay, Weitz, et al., 2022). Patients who had ultrasonography largely did so to assess pregnancy duration; a small proportion of patients obtained ultrasonography owing to concerns about ectopic pregnancy, which is consistent with clinical recommendations. Most participants who had ultrasonography before telehealth abortion obtained it at a clinic, emergency room, or hospital.

In our bivariate analyses, participants who were younger than 25 years of age, unemployed, had less than a 4-year degree, or were food insecure were more likely to obtain ultrasonographic imaging. In our multivariable analysis, we found that younger participants, participants who were food insecure, and those with longer pregnancy durations were more likely to obtain screening ultrasonography. Patients who are further out from their last menstrual period may be less certain about their pregnancy duration and thus may require ultrasonography to confirm their pregnancy is under the clinic’s gestational limit for telehealth abortion. Younger individuals have been found to identify their pregnancy duration at later gestations, although we found an independent effect of age after controlling for pregnancy duration. Overall, individuals present for care later in pregnancy for a variety of reasons, including decisional uncertainty, delayed recognition of pregnancy, and not having the money to pay for the abortion (Ralph et al., 2022; Upadhyay, Weitz, et al., 2022). This delay may result in the need for ultrasonographic imaging at the time of pregnancy identification to determine the duration of the pregnancy. Subsequent research should assess whether no-test medication abortion clinical screening criteria disproportionately divert members of groups facing systemic barriers to pre-abortion ultrasonography, causing such groups to become further delayed.

These findings suggest that individuals who are more vulnerable to financial instability have higher likelihoods of obtaining ultrasonography. Given these findings, the absence of an association between ultrasonography and insurance type, specifically Medicaid and being uninsured, was unexpected. Further research on this topic could help shed light on these associations.

Although the majority of participants obtained ultrasonography in a hospital, clinic, or emergency room, the use of crisis pregnancy centers to obtain ultrasonography among 14% of the sample who obtained it is an important finding of this study. Research has suggested that visits to crisis pregnancy centers are more common among Black, non-Hispanic patients and patients with the fewest resources (Van Ryn & Burke, 2000). Crisis pregnancy centers provide free or low-cost imaging services, which may partially explain why pregnant people of low socioeconomic status are more likely to obtain imaging at these locations. Despite being accessible, crisis pregnancy centers are known to provide false or dishonest information to pregnant people regarding the risks of abortion (Cartwright et al., 2021; Rice et al., 2021). This finding points to a need for additional abortion-supportive sources for timely, no-cost pre-abortion ultrasonography that does not create situations where patients may experience additional bias from providers (Aiken et al., 2024; Bryant et al., 2014).

This study is not without limitations. We may have underestimated patients’ use of crisis pregnancy centers for preabortion ultrasonography if patients mistakenly identified crisis pregnancy centers as health centers or clinics. This factor may have resulted in overly conservative estimates of how often telehealth patients obtain ultrasonography at crisis pregnancy centers. Similarly, there may be reporting errors in other locations where they received ultrasonographic imaging. Additionally, participants who were referred for ultrasonography by virtual clinic providers and then did not follow up for abortion from their telehealth providers were not included; the characteristics of these patients may have been different than the characteristics of the patients who were included in our analysis. This factor may impact the generalizability of these findings. Last, we were unable to distinguish between participants who preferred to obtain ultrasonographic imaging versus those who were required to do so for a medical reason. Our use of electronic medical record data offered insights into whether provider referral was provided to our participants, but discerning this information directly would be a valuable next step for future studies.

### Implications for Practice and/or Policy

Pre-abortion ultrasonography is not needed for most patients obtaining telehealth medication abortion care. As telehealth services for abortion care expand, we must understand patient needs and preferences for ultrasonography. This study suggests that a minority of patients obtain pre-abortion ultrasonography. Providers of abortion care who may be considering offering telehealth may benefit from collaborating with free, timely, abortion-supportive ultrasonography facilities for those who are not confident about their pregnancy duration. This study also suggests that patients may receive ultrasonography owing to interaction with crisis pregnancy centers, which act as barriers to care for patients seeking abortion care (Kavanaugh et al., 2019). It is essential that telehealth providers have information on available sources for ultrasonography that are convenient and affordable for patients. Some people who seek abortion will always have a need for ultrasonography, whether medically indicated or not. Providers can work to prioritize accessible ultrasonography options that are low cost, covered by insurance, high quality, and geographically accessible to mitigate barriers and increase convenience for patients.

## Figures and Tables

**Table 1:** Characteristics of Telehealth Abortion Patients From the CHAT Study Who Completed the First Survey (*n* = 1,775)

Characteristics	Total Sample (*n* = 1,775)	Pre-abortion Ultrasonography (*n* = 194 [10.9%])	No Pre-abortion Ultrasonography (*n* = 1,581 [89.1%])	χ^2^, *p* Value*
Age				.003^†^
<18 years	8 (0.5)	2 (1.0)	6 (0.4)	
18–24 years	454 (25.6)	68 (35.1)	386 (24.4)	
25–34 years	837 (47.2)	87 (44.8)	750 (47.4)	
≥35 years	335 (18.9)	23 (11.9)	312 (19.7)	
Missing	141 (7.9)	14 (7.2)	127 (8.0)	
Race or ethnicity				.14^†^
Asian, Native Hawaiian, or Pacific Islander	118 (6.6)	11 (5.7)	107 (6.8)	
Black or African American	161 (9.1)	20 (10.3)	141 (8.9)	
Hispanic or Latinx	233 (13.1)	30 (15.5)	203 (12.8)	
American Indian or Alaska Native, Middle Eastern or North African	24 (1.4)	0 (0.0)	24 (1.5)	
White	922 (51.9)	89 (45.9)	833 (52.7)	
Multiracial	242 (13.6)	32 (16.5)	210 (13.3)	
Unknown	75 (4.2)	12 (6.2)	63 (4.0)	
Current student				.24^†^
No	1,297 (73.1)	139 (71.6)	1,158 (73.2)	
Yes, in school full time	297 (16.7)	30 (15.5)	267 (16.9)	
Yes, in school part time	147 (8.3)	23 (11.9)	124 (7.8)	
Missing	34 (1.9)	2 (1.0)	32 (2.0)	
Highest level of education achieved				6.4, .040
High school or less	301 (17.0)	35 (18.0)	266 (16.8)	
Some college, some technical school, or associate’s degree	742 (41.8)	95 (49.0)	647 (40.9)	
Completed 4-year degree or more	732 (41.2)	64 (33.0)	668 (42.3)	
Employment status				7.19, .028
Unemployed	338 (19.0)	44 (22.7)	294 (18.6)	
Employed	1,413 (79.6)	144 (74.2)	1,269 (80.3)	
Unknown	24 (1.4)	6 (3.1)	18 (1.1)	
Food insecurity				13.07, .001
Never	1,192 (67.2)	108 (55.7)	1,084 (68.6)	
Sometimes or often	532 (30.0)	78 (40.2)	454 (28.7)	
Unknown	51 (2.9)	8 (4.1)	43 (2.7)	
Prior pregnancies				.707^†^
No	701 (39.5)	81 (41.8)	620 (39.2)	
Yes	1,070 (60.3)	113 (58.2)	957 (60.5)	
Unknown	4 (0.2)	0 (0.0)	4 (0.3)	
Prior ectopic pregnancy				.804^†^
No prior ectopic pregnancy	1,630 (91.8)	180 (92.8)	1,450 (91.7)	
Prior ectopic pregnancy	1 (0.1)	0 (0.0)	1 (0.1)	
Unknown or not asked	144 (8.1)	14 (7.2)	130 (8.2)	
Prior medication abortion				.656^†^
No	1,219 (68.7)	139 (71.6)	1,080 (68.3)	
Yes	506 (28.5)	51 (26.3)	455 (28.8)	
Unknown or missing	50 (2.8)	4 (2.1)	46 (2.9)	
Prior miscarriage				2.88, .237
No	1,108 (62.4)	113 (58.2)	995 (62.9)	
Yes	181 (10.2)	26 (13.4)	155 (9.8)	
Unknown	486 (27.4)	55 (28.4)	431 (27.3)	
Pregnancy duration at abortion intake				93.49, <.001
<35 days	447 (25.2)	16 (8.2)	431 (27.3)	
35–49 days	884 (49.8)	89 (45.9)	795 (50.3)	
50 days or more	266 (15.0)	71 (36.6)	195 (12.3)	
Unknown	178 (10.0)	18 (9.3)	160 (10.1)	
Health insurance coverage				.116^†^
Private insurance	1,046 (58.9)	101 (52.1)	945 (59.8)	
No insurance	347 (19.5)	45 (23.2)	302 (19.1)	
Medicaid	328 (18.5)	44 (22.7)	284 (18.0)	
Unknown	54 (3.0)	4 (2.1)	50 (3.2)	

Values are number (%).

* *p* values are from χ^2^ tests except where indicated.

† *p* value corresponds with a Fisher’s exact test. Fisher’s exact test does not produce a test statistic value, so only a *p*-value is shown.

**Table 2:** Factors Associated With Having Screening Ultrasonography From Multivariable Logistic Regression (*n* = 1,775)

Characteristics	Adjusted Odds Ratio (95% CI)
Age (years)*	
<25	Reference
25–34	0.65^†^ (0.044–0.96)
≥35	0.42^‡^ (0.25–0.72)
Race or ethnicity^§^	
White	Reference
Black or African American	1.17 (0.68–2.03)
Hispanic or Latin	1.19 (0.74–1.90)
Asian/other/unknown	1.12 (0.77–1.63)
Education	
High school or less	Reference
Some college, some tech school, or associate’s degree	1.50 (0.96–2.34)
Completed 4-year degree or more	1.29 (0.78–2.12)
Employment	
Unemployed	Reference
Employed	0.80 (0.55–1.19)
Unknown	2.87^†^ (1.01–8.15)
Food Insecurity	
Never	Reference
Sometimes/often	1.51^†^ (1.08–2.10)
Unknown	1.67 (0.73–3.85)
Prior pregnancies^¶^	
None or unknown	Reference
Yes	1.13 (0.76–1.69)
Prior medication abortion	
No	Reference
Yes	0.84 (0.56–1.27)
Unknown	0.78 (0.27–2.27)
Pregnancy duration	
<35 days	Reference
35–49 days	2.98^‖^ (1.72–5.17)
50+ days	9.48^‖^ (5.32–16.87)
Unknown	4.42^‖^ (2.03–9.60)
Health insurance^#^	
Private	Reference
None or unknown	0.97 (0.65–1.44)
Medicaid	1.19 (0.78–1.80)

* For Age, participants under 18 and between the ages of 18–24 were grouped into the <25 group owing to small sample sizes.

† *p* < .05.

‡ *p* < .01.

§ For race or ethnicity, unknown was grouped with Asian and other owing to small numbers.

¶ For prior pregnancies, none and unknown were grouped together owing to small numbers.

‖ *p* < .001.

# For health insurance, none and unknown were also grouped together owing to small numbers.
